# Effective team-based primary care: observations from innovative practices

**DOI:** 10.1186/s12875-017-0590-8

**Published:** 2017-02-02

**Authors:** Edward H. Wagner, Margaret Flinter, Clarissa Hsu, DeAnn Cromp, Brian T. Austin, Rebecca Etz, Benjamin F. Crabtree, MaryJoan D. Ladden

**Affiliations:** 1 MacColl Center for Health Care Innovation, Group Health Research Institute, 1730 Minor Ave., Suite 1600, Seattle, WA 98101 USA; 2 Community Health Center, Inc., Middletown, CT USA; 3 Center for Community Health and Evaluation, Group Health Research Institute, Seattle, WA USA; 4 Department of Family Medicine, Virginia Commonwealth University, Richmond, VA USA; 5 Department of Family Medicine and Community Health, Rutgers-Robert Wood Johnson Medical School, Piscataway Township, NJ USA; 6 Robert Wood Johnson Foundation, Princeton, NJ USA

**Keywords:** Primary care, Practice team, Patient-centered medical home

## Abstract

**Background:**

Team-based care is now recognized as an essential feature of high quality primary care, but there is limited empiric evidence to guide practice transformation. The purpose of this paper is to describe advances in the configuration and deployment of practice teams based on in-depth study of 30 primary care practices viewed as innovators in team-based care.

**Methods:**

As part of LEAP, a national program of the Robert Wood Johnson Foundation, primary care experts nominated 227 innovative primary care practices. We selected 30 practices for intensive study through review of practice descriptive and performance data. Each practice hosted a 3-day site visit between August, 2012 and September, 2013, where specific advances in team configuration and roles were noted. Advances were identified by site visitors and confirmed at a meeting involving representatives from each of the 30 practices.

**Results:**

LEAP practices have expanded the roles of existing staff and added new personnel to provide the person power and skills needed to perform the tasks and functions expected of a patient-centered medical home (PCMH). LEAP practice teams generally include a rich array of staff, especially registered nurses (RNs), behavioral health specialists, and lay health workers. Most LEAP practices organize their staff into core teams, which are built around partnerships between providers and specific Medical Assistants (MAs), and often include registered nurses (RNs) and others such as health coaches or receptionists. MAs, RNs, and other staff are heavily involved in the planning and delivery of preventive and chronic illness care. The care of more complex patients is supported by behavioral health specialists, RN care managers, and pharmacists. Standing orders and protocols enable staff to act independently.

**Conclusions:**

The 30 LEAP practices engage health professional and lay staff in patient care to the maximum extent, which enables the practices to meet the expectations of a PCMH and helps free up providers to focus on tasks that only they can perform.

**Electronic supplementary material:**

The online version of this article (doi:10.1186/s12875-017-0590-8) contains supplementary material, which is available to authorized users.

## Background

The coordinated involvement of teams in patient care has emerged as an essential feature of patient-centered medical homes (PCMHs) capable of achieving the quadruple aim—improving patient health, enhancing patient experience, reducing health care costs, and improving the work life of providers and staff [1, 2]. Clinical trials show that the involvement of medical assistants, nurses and other staff in delivering clinical services to diabetic, hypertensive, and depressed patients leads to substantial improvements in disease control [3–7]. Qualitative studies of exemplary primary care practices conclude that practices without effective teams have difficulty performing functions such as population management, care coordination and care management that are critical to the success of a PCMH [2, 8, 9]. If patients experience care by well-organized teams, both satisfaction with overall care and with their provider increase [10, 11]. Lastly, recent studies indicate that more effective teams are associated with less job exhaustion and burnout for physicians and other staff [12, 13].

Despite the growing recognition of the importance of teams, there is relatively little empiric evidence to guide practices wanting to create high-performing primary care teams. In response, the Robert Wood Johnson Foundation created The Primary Care Team: Learning from Effective Ambulatory Practices (PCT-LEAP), a national program devoted to helping primary care practices develop more effective primary care teams using insights and examples gleaned from the study of innovative, high-performing practices [14]. In an earlier paper, we described preliminary findings on the changing roles of key staff across LEAP practices [14]. Since the publication of that paper, we have completed the site visits to all 30 practices, distilled key themes from the data, developed a web-based technical assistance program (www.improvingprimarycare.org), and used the program to help practices implement team-based primary care. In this paper, we describe major themes in team configuration and team-based care observed in LEAP practices, and highlight specific changes in staff composition or deployment that appear to be promising departures from traditional practice.

## Methods

### Setting

LEAP is co-directed by EW and MF, and is based at the MacColl Center for Health Care Innovation. A National Advisory Committee (NAC) has played important roles in the selection of practices, conduct of site visits, and development of the technical assistance program.

### Site selection

Practice selection involved a multi-stage process comprised of: first, nomination by national primary care experts of primary care practices that creatively use their workforce to improve care quality and efficiency; second, data collection from nominated practices; and finally, data review and grading. From a literature review of primary care workforce innovations [15], we identified 50 thought leaders and asked them to recommend potentially relevant practices and additional nominators. Ultimately, we elicited names of 435 practices from 387 individuals. Roughly ½ of the nominated organizations were eliminated, most commonly because they were not primary care. One of the authors (RE) completed 45 min telephone interviews with a leader in the remaining 227 practices. Information collected included: practice organization and size, patient characteristics, payment mix, workforce innovations, practice team composition and roles; and practice performance and quality improvement. The descriptive and interview data from each practice were summarized in a two-page structured report for 154 practices deemed to have workforce innovations.

Four NAC members and staff independently reviewed each two-page report and rated the practice team innovations. We then requested available data on quality of care and patient experience from the 70 highest rated practices. Based on the practice team innovativeness ratings and the review of performance data, the NAC selected 30 practices that reflect a range of geographic locations/settings, practice organizational types, and populations served. Within each multi-site organization, data collection focused on a single practice site where we could best observe high functioning practice teams. Table 1 describes the 30 LEAP practice sites, which comprise 15 Federally Qualified Health Centers (FQHCs) including a nurse-managed center, and 15 private practices, nine of which were part of multi-specialty groups or integrated systems. Four sites are major medical residency training sites, and one serves as a Nurse Practitioner residency site. Most (77%) LEAP sites are part of multi-site practice organizations. Panel sizes per primary care provider range from 800 to 2750. Panel sizes tend to be larger in private practices with smaller Medicare populations. Twenty-one of the 30 sites were NCQA level 3 PCMH’s and 3 more had state-based PCMH certification. Most LEAP private practices primarily depend on fee-for-service reimbursement from commercial plans, Medicare, and Medicaid. FQHCs largely rely on Medicaid and their federal grant. Only one LEAP site reported that the majority of their revenue was from capitation.

**Table 1:** Characteristics of LEAP Practice Sites

Organizational Type	
Federally Qualified Health Center	15 (50%)
Private Practice	
Multi-specialty Group	9 (30%)
Primary Care	6 (20%)
Residency Training Program	
Medical	4 (13%)
Nurse Practitioner	1 (3%)
No residency	25 (83%)
Region	
New England (MA, ME,NH)	5 (17%)
Middle Atlantic (NY, PA,WV,DC)	7 (23%)
Southeast (SC,LA)	2 (7%)
Midwest (OH,IN,IA,WI,MN)	5 (17%)
Southwest (TX,NM,CO)	5 (17%)
Pacific Coast (CA,OR,WA)	6 (20%)
Location	
Urban	9 (30%)
Suburban	8 (27%)
Rural	11 (37%)
Multiple	2 (7%)
Number of Sites in the Practice Organization	
One	7 (23%)
2–5	6 (20%)
6–10	8 (27%)
11–20	4 (13%)
21+	5 (17%)
Panel Size per full-time PCP	
< 1500	10 (33%)
1500–1999	9 (30%)
2000–2500	10 (33%)
> 2500	1 (3%)
PCMH Certification	
NCQA level 3	20 (67%)
JCAHO	1 (3%)
State Certification	3 (10%)
No PCMH Certification	6 (20%)

We sought to compare the staffing of LEAP practices with less highly selected American primary care practices. A recent paper described the staffing of 496 practices in seven regions of the U.S. that participated in the Centers for Medicare and Medicaid Services Comprehensive Primary Care Initiative (CPC) [16]. Federally Qualified Health Centers and Rural Health Clinics were excluded from CPC.

### Site visits

Each of the 30 LEAP practice sites hosted a 3-day site visit by a team of 3–5 individuals that included a clinician investigator, a qualitative researcher, and a research assistant. All site visitors received training in qualitative data collection. Site visit activities were generally limited to the single practice location described above. The site visits included: tours of clinic space and the EMR; formal semi-structured (recorded and transcribed) and informal interviews with practice leaders, providers, and staff; staff and patient shadowing; observations of huddles and other team meetings; a staff survey; and collection of photographs, practice materials and tools. Site visits took place between August 2012 and September 2013. Guides for interviews conducted and observations made during site visits are listed in the Additional files 1, 2 and 3.

### Data analysis

We used a modified Delphi process with all site visitors to identify advances in team configuration and roles observed during site visits [17]. We verified these advances and related themes with LEAP sites through an in-person convening of representatives from all sites. We used Atlas.ti to help organize and store the qualitative data from site visits and the in-person meeting of practice representatives. We developed codes to capture the key domains of interest, which were influenced by the findings from the Delphi process and the in-person meeting. We coded site visit interview transcripts, site visitor notes and summaries, field notes from shadowing staff and patients, staff survey comments, and notes from the in-person meeting. Coded segments were then reviewed and themes identified and confirmed.

### Technical assistance

We summarized major themes and related findings from the study of LEAP practices in a web-based technical assistance program to help other primary care practices build and deploy effective teams. The website--“Primary Care Team Guide” (www.improvingprimarycare.org) --became publicly available in October 2014. Since its release, the Primary Care Team Guide has supported technical assistance for developing effective teams in three different learning collaboratives involving over 60 primary care practices and a national webinar series.

## Results

Table 2 describes the major advances in team-based care identified by site visitors and confirmed by LEAP practice leaders and qualitative data analysis. It includes both major trends across sites and less common innovations with the potential to substantially change traditional practice.

**Table 2:** Primary Care Staff Organization, Roles and Activities in LEAP Practices

Innovation Area	Major Trends	Promising Innovations
Primary care team structure	• Providers and their panels are supported by a core team built around strong provider-MA partnerships. • Multi-provider core teams often include, RNs, and front desk staff. • Core team members including PCPs share offices and work spaces. • Extended practice teams often include RN care managers, behavioral health specialists, and pharmacists.	• Each PCP works with 2 MAs, who remain with each patient throughout their visit—doing intake, scribing for the PCP, and handling post-visit questions and issues.
Enhanced role of medical assistants	• MAs review charts of scheduled patients and lead core team huddles to plan care. • MAs arrange or deliver most preventive care procedures. • MAs often involved in outreach to patients with care gaps or needing follow-up. • MAs are actively involved in Quality Improvement and play leadership roles.	• MAs with additional training in self-management support and diabetes care conduct individual and small group visits with diabetic patients.
Roles of Registered Nurses	• Core team RNs provide follow-up care, skills training, and self-management support to chronically ill patients in nurse encounters or conjoint visits. • Team RNs use nurse visits and standing orders to manage common acute illnesses. • RN care managers work with small panels of high risk patients.	• RNs use delegated order sets to titrate medications for patients with common chronic conditions—e.g., warfarin, anti-hypertensive drugs.
Layperson Patient Care Roles	• Laypersons help patients address needs for information, community resources, and coordination of their care.	• Laypersons trained in self-management counseling serve as health coaches. • Layperson EMR experts make changes to the EMR supportive of quality improvement.
Managing Complex Illness	• RN Care Managers work with small panels of sicker patients, including those discharged from hospital. • Behavioral Health Specialists, other social workers, and lay care coordinators/community health workers address psychosocial needs. • Pharmacists provide Medication Therapy Management services to multi-problem patients.	• Weekly or bi-weekly case conferences convene multi-disciplinary clinic staff to discuss challenging patients and develop a comprehensive care plan, and review progress of previously discussed patients.
Behavioral Health Integration	• Core team (MAs and RNs) involved in depression screening and follow-up. • On-site Behavioral Health Specialists facilitate warm handoffs and provide short-term therapy and crisis management. • Advice on psychotropic drugs is obtained from on-site or consulting Psychiatrists or Psychiatric NPs.	• Patients on chronic opioid therapy are tracked, asked to sign contracts, and offered in-clinic buprenorphine therapy if warranted.
Clinic-Community Connections	• Practices hire staff from populations served by the clinic. • Designated practice team members help patients identify and access community services. • Practice actively cultivates partnerships with community organizations to address social and environmental issues.	• The practice works with other agencies in the community to address social determinants of health.

### Team structure

The primary care staff in LEAP sites is generally divided into: smaller core teams that work with specific providers and their panels; and an extended team that serves all providers and patients at the site. LEAP core teams are generally built around a single or small group, sometimes called a pod of stable Provider—Medical Assistant partnerships. Table 3 shows the composition of core teams, as defined by practice leaders at each site. Core teams in a large majority of LEAP sites include multiple provider/MA dyads, and often include registered nurses (RNs) and other staff. Licensed practical nurses (LPNs) on core teams are most commonly used in MA roles and included with MAs in Table 3. In one-third of practices, front desk staff works with specific core teams, are encouraged to develop personal relationships with patients, and help collect and collate patient information prior to visits. In 5 practices with high psychosocial morbidity, Behavioral Health Specialists are members of core teams.

**Table 3:** Core Team Composition in LEAP Practices: Number and Percentage of Practices

	1 Primary Care Provider* *n* = 6	2-3 Primary Care Providers* *n* = 15	4+ Primary Care Providers* *n* = 9	All Practices *n* = 30
Medical Assistants**	6 (100%)	15 (100%)	9 (100%)	30 (100%)
Registered Nurses	1 (17%)	6 (40%)	7 (78%)	14 (47%)
Licensed Practical Nurses (LPNs)	0	4 (27%)	0	4 (13%)
Front Desk Staff	1 (17%)	8 (53%)	1 (11%)	10 (33%)
Behavioral Health	0	3 (20%)	2 (22%)	5 (17%)
Health Coach	1 (17%)	1 (7%)	2 (22%)	4 (13%)
Lay Care Coordinator	0	1 (7%)	1 (11%)	2 (7%)
Social Worker	0	0	1 (11%)	1 (3%)

*Number of Paneled Providers (MD, DO, ND, NP, PA) on each core team

**Includes LPNs if used as Medical Assistants

The ratio of MAs to providers is 1:1 in most practices, but 6 LEAP practices link multiple MAs (1.5–3 MAs) with each provider. One LEAP practice assigns 2 MAs to each provider, which allows one MA to remain with each patient throughout their clinic visit. The MA enters the provider’s description of the findings and plan into the EHR (often called scribing) [18], while the other MA is checking in the next patient. The innovation is being disseminated throughout the practice, as a pilot test demonstrated that PCPs were more satisfied AND could see more patients.

LEAP extended teams often include RN care managers, BHSs, and Pharmacists who support all core teams, either working with a subpanel of patients or by referral from the core team. Table 4 compares the availability of various staff roles in LEAP sites (on both core and extended teams) with 496 private practices (FQHCs were excluded) selected for the Centers for Medicare and Medicaid Services Comprehensive Primary Care Initiative (CPC) [16]. We classified RNs in LEAP practices either as team RNs or care managers depending on their primary functions. RN care managers were more prevalent in LEAP practices than in CPC practices. Pharmacists and social workers were much more available in LEAP practices (37 and 63%, respectively) than in CPC practices (7 and 5%, respectively). Most social workers in LEAP practices are licensed therapists and function as BHSs. Table 4 also shows some differences in staffing between LEAP private practices and FQHCs of similar size with the latter less likely to employ RN care managers and more likely to employ BHSs.

**Table 4:** Percentage of CPC and LEAP Practices with Different Types of Staff

	<5 Primary Care Providers*		5+ Primary Care Providers*		
	CPC *N* = 364	LEAP Private Practices *N* = 3	CPC *N* = 132	LEAP Private practices *N* = 12	LEAP FQHCs *N* = 15
Administrative staff	98	100	100	100	100
Medical assistants	86	100	95	100	100
Team Registered Nurses	29	33	54	50	53
Registered Nurse Care managers	20	33	36	75	33
Pharmacists	4	0	17	42	40
Social Workers	2	33	13	58	73
Nutritionists	3	50	8	20	14

*Number of providers in the practice site

### The enhanced role of medical assistants

LEAP practices encourage MAs to develop personal relationships with patients and be more involved in patient care [2, 19]. Role expansion generally includes the pre-visit review of the charts of scheduled patients (chart-scrubbing) to identify needed preventive and chronic care services. These “care gaps” are discussed in pre-session huddles with their provider partner(s) and the visit planned. MAs deliver or arrange many preventive services by standing orders before the provider enters the examination room. Between visits, they make phone calls to check on recently seen patients. In some LEAP practices, MAs receive additional training in chronic disease management and self-management counseling and act as health coaches [20, 21]. In one practice, diabetes control improved when MAs trained in self-management support and diabetes care started meeting individually or in small groups with diabetic patients.

### The changing roles of Registered Nurses (RN)

There was considerable variation among LEAP practices in RN roles. One-half of LEAP sites had designated RN care managers who work with sicker, more complex patients outside of clinic visits (See Complex Care Management below). RN role variability was predominantly seen among those working on or with core teams in the care of patients visiting the clinic. While team RNs in some LEAP sites are mostly responding to patient phone calls, doing procedures, and teaching patients, RNs in a number of LEAP practices are providing direct patient care via independent nurse visits, conjoint visits with a provider, or electronic communications. These nurses play major roles in the routine management of patients with chronic illness including teaching and follow-up [22]. RNs in 13 practices use delegated order sets to manage patients on warfarin, and in a few practices RNs titrate anti-hypertensive and hypoglycemic medications. Some practices also use independent nurse visits and standing orders to provide care for a variety of common acute illnesses.

### The growing involvement of lay roles in patient care

One-half of LEAP practices employ staff without formal health training or certification (lay roles) in patient-facing roles such as health coach, care navigator/coordinator, or community health worker. In 20% of LEAP practices, lay staff are important members of core teams. These layperson roles tend to fall into two categories: health coaching or self-management support [20, 21, 23, 24] and/or assisting in patient follow-up or care coordination [25, 26]. Other lay roles help patients obtain specialist consultations or community resources.

One-half of LEAP practices employed health coaches. While the majority of health coaches were MAs or nurses, some were laypersons trained in self-management counseling. A couple of LEAP sites have hired computer-savvy laypersons who receive intensive training in the functionality and adaptability of the practice’s EMR. Their primary role is to work closely with quality improvement teams and care teams to ensure that the EMR reflects and supports new care processes.

### Closing the loop

During site visits, we frequently heard staff talk about “closing the loop” on gaps or failures in care—e.g., poorly controlled chronically ill patients without appointments, or preventive services that are overdue. To close these loops, LEAP practices have exploited or developed data systems that enable them to track critical patient information. In most LEAP sites, multiple members of the practice team routinely review data on different patient populations and reach out to those needing further care. For example, patients recently hospitalized are tracked by RN care managers while MAs or panel managers look for patients overdue for preventive care or chronic disease follow-up.

A few LEAP practices have created a referral manager/coordinator position to help patients access specialty and other community services, and ensure that all parties receive the information they need [27].

### Managing complex illness

Essentially all LEAP practices have made the care of patients with multiple health problems including recent hospital discharges a priority, and altered the composition and functioning of their practice teams accordingly. One-half of practices have RN care managers. These nurse care managers sometimes collaborate with other staff knowledgeable about community resources to better address the psycho-social needs of patients. 40% of LEAP practice teams have pharmacists, most of whom work with patients on complex drug regimens.

A few practices regularly review complex patients in multi-disciplinary case conferences. In one FQHC, the entire clinic staff meets weekly to discuss how they might better care for 1 or 2 of their most challenging patients.

### Behavioral health integration

Nineteen LEAP practices (63%) employ one or more BHSs who consult with the team about mental health, substance abuse, and other behavioral health issues, and provide short-term crisis management and therapy. Most commonly in LEAP practices, the BHS is a Licensed Clinical Social Worker. A few practices have added psychiatrists or psychiatric nurse practitioners to consult on psychoactive drugs. Multiple team members participate in providing mental health care-- e.g., MAs perform depression screening before the provider enters the room, and RNs or BHSs make follow-up phone calls to depressed patients started on treatment.

Most LEAP practices are addressing the opioid epidemic in various ways. Patient contracts, urine drug testing, and medication monitoring to control drug-seeking behavior are common. A handful of practices with interested providers have successfully instituted buprenorphine treatment programs.

### Clinic-community connections

A long-term relationship with and commitment to their community draws many health professionals and staff to primary care. Among LEAP practices, this community focus manifests in different ways. Many try to hire staff representative of their patient populations to help them communicate with their patients and understand the cultural and environmental issues that may be influencing their health and treatment. Some, including a few private practices, provide or collaborate with other agencies to provide programs that reach beyond their patients to support the health of the broader community such as healthy eating and active living activities, community gardens, etc. For example, a LEAP site in a low-income rural community was the driving force behind the community’s efforts to reduce childhood obesity.

## Discussion

The Affordable Care Act makes clear that American primary care must be strengthened and improved through transformation of existing practice organizations to PCMHs [28]. By most definitions of a PCMH, practices are expected to provide comprehensive, high quality care in the office, use data to identify and eliminate gaps in care, track and support patients between office visits, and address comprehensive patient needs including behavioral health issues [1, 2, 29]. The innovative, high performing LEAP practices have expanded the roles of existing staff and added new personnel to provide the person power and skills needed to achieve the quadruple aim and perform the tasks and functions expected of a PCMH [1, 2, 30–32].

Recent policy-maker enthusiasm for strengthening the primary care sector stems in part from the expectation that high cost patients, generally those with multiple chronic conditions, can be effectively managed in the community. In response, many LEAP practices either employ or have access to RN care managers, BHSs, and Pharmacists to augment services to high risk patients. While LEAP practice teams include a somewhat broader array of disciplines than teams in more traditional practices, the expanded roles of traditional staff such as MAs or RNs and their involvement in care delivery probably has the largest impact on care quality. To sustain role expansion, staff are being continuously trained, mentored, and assessed. Workflows are documented and incorporated into staff training. Several LEAP practices have modified their EMR to support new and improved workflows. In addition, widespread use of protocols and standing orders increases efficiency by allowing staff to work independently.

In addition to the general trends in staffing described above, we saw promising advances in staffing and care delivery with the potential to fundamentally change primary care practice [33]. For example, with additional training and support: MAs or laypersons can take on much of the responsibility for documenting encounters or providing self-management support; RNs can titrate medications for common illnesses; and laypersons can substantially improve care coordination or the utility of the EMR.

Practice leaders reported that the largest barrier to the development of effective practice teams was dependence of the practice on fee-for-service reimbursement because of the premium placed on provider involvement in care. Bundled or capitated payments facilitate shifting clinical tasks from providers to other staff. While LEAP practice leaders generally feel that they are aligning their organizations with future payment reforms, most are currently relying on grant funds or negotiated arrangements with local health insurers to support workforce innovation.

The major limitations of LEAP and this paper are the reliance on cross-sectional data collected from a limited number of highly selected practices 3–4 years ago. Site selection involved a rigorous process that looked for practices that demonstrated workforce innovation, quality improvement infrastructure, and quality of care. Although we reviewed clinical performance data collected by each practice, the variability in the content and quality of measurement from site to site limited our confidence in attesting to the quality of care provided. However, direct observations of care delivery in the 30 practices confirmed that these were high performing practices. In selecting practices for LEAP, we sought diversity in geography, business models, and patient populations served. While the practice diversity potentially made data interpretation more difficult, the trends in the configuration and deployment of practice teams described above were shared across the range of practices and bridged key distinctions such as between FQHC’s and private practices. We have engaged leaders from LEAP sites in our technical assistance activities over the years since the site visits, which gives us some assurance that the observations presented above reflect current practice. Our recent involvement in practice transformation efforts focused on building more effective primary care teams suggests that the advances in team-based care described above are not widespread.

Primary care teams are complex adaptive systems [34], consisting of interdependent and constantly changing parts. As a consequence, it will be very difficult to empirically test each of the many moving parts. Nonetheless, we urgently need comparative trials to help clarify the optimal structure and functioning of cost-effective teams capable of providing the comprehensive, high quality services expected of PCMHs. In the absence of data from rigorous trials, innovative, high quality practices are perhaps the best source of information and ideas on which to base efforts to transform primary care practices.

## Conclusion

To build more effective practice teams, the 30 LEAP practices have expanded the roles of existing staff and added new staff and new competencies. In particular, MAs play important patient care roles such as delivering preventive care or health coaching, and RNs are more involved in face-to-face care delivery. Most practices have developed in-house training activities since external training programs for key roles such as MAs or RNs generally aren’t preparing trainees for these expanded roles. Key new competencies include behavioral health, RN care management, and pharmacy. These richer, more effective teams enable practices to achieve high levels of performance and helps free up providers to focus on tasks that only they can perform.

## Additional files

Additional file 1: LEAP Interview Guide – Clinic Staff. Guide for conducting recorded interviews with various staff members in each visited clinic. (DOCX 21 kb)
Additional file 2: LEAP Interview Guide – Leadership. Guide for conducting recorded group interviews with administrative and clinical leaders in each visited clinic. (DOCX 22 kb)
Additional file 3: PCT LEAP Site Visit Observation Guide – Clinical Expert. The guide includes instructions as to what clinical site visitors should observe and document during site visits. (DOC 125 kb)

## Abbreviations

BHS: Behavioral health specialist
CPC: Comprehensive primary care initiative
EMR: Electronic medical record
FQHC: Federally qualified health center
LPN: Licensed practical nurse
MA: Medical assistant
NAC: National Advisory Committee
NCQA: National Committee on quality assurance
PCMH: Patient-centered Medical Home
PCP: Primary care provider
PCT-LEAP: Primary care team-learning from effective ambulatory practices
RN: Registered nurse
