# Supplementary material for: Effective team-based primary care: observations from innovative practices
Source: BMC Fam Pract. 2017 Feb 2;18:13. doi: 10.1186/s12875-017-0590-8 (PMC5289007; doi:10.1186/s12875-017-0590-8)
Supplement: Additional file 1: — LEAP Interview Guide – Clinic Staff. Guide for conducting recorded interviews with various staff members in each visited clinic. (DOCX 21 kb) [file 12875_2017_590_MOESM1_ESM.docx]

**PCT-LEAP Site Visit Interview Guide**

**Front Line Staff (1 hour)**

04-16-13

1 hour

*Thank you for participating in this interview. Do you have any questions about this or the LEAP Project?*

<Collect signed consent form – not a research study but we do want to publish>

1. **Role**
2. Can you please say your title and briefly describe your role within <org name>?
   1. How long have you been at this practice? How has your role changed over time?
   2. What other kind of practices did you work at (or other healthcare background) before coming to this practice?
   3. What training did you receive to orient you to your role here?
3. **Organization History/Mission/Evolution**
4. Can you take a few minutes to briefly describe your organization’s history?
   1. How long has your organization been in existence?
   2. How would you describe the leadership structure? (might want to specifically ask about clinical medical leadership: is there a CMO? ACMO? CNO? Chief of behavioral health? Do all medical staff report through the CMO and then to CEO?)
   3. Can you tell me how are key strategic decisions made? (might want to add in areas such as growth, change in staffing patterns, or change in services)
   4. Is there anyone who has left the organization that was particularly important to its history and/or the development of the innovations we are interested in? If so, who was that person and what role did they play in making the organization what it is today?
   5. Can you please tell me about any key turning points/milestones in your organization’s history that you believe are critical to understanding the organization now?
5. Can you please describe your organization’s mission?
6. How would you describe your target population(s)?
   1. [If the org’s mission is to serve a specific population] How has your organization stayed responsive to the needs of this population over time?
7. What other community or contextual factors do you think have been critical to shaping <org name>?
   1. What other types of support for your work are available in your community?
   2. What unique challenges do you face in this community?
   3. How does your state’s Medicaid program impact your practice?
   4. Please tell me about any specific issues in your state or community that impact billing and reimbursement.
   5. How do state policy impact staffing (such as scope of practice restrictions, restrictions on MA practice)?
8. What are the overarching goals or principles that drive major decisions at this clinic?
9. What is your organization’s approach to quality improvement – or another way to think about what we are getting at, how do you *make change*?
   1. Do you have a particular model for QI that you follow?
   2. What structures are in place to support QI efforts?
   3. Can you describe your performance improvement plan? <if necessary> Do you have a written performance improvement plan?
   4. How are staff members involved in QI? How have you been involved?
10. **Primary Care Workforce Innovation**
11. Can you tell us how your teams are organized and what your role is within that team?
    1. Are there any other unique features of your care teams that we should be aware of?
    2. How do you feel about your current job description or role?
    3. What happens when the clinic is very busy and you begin to run behind? What adjustments are made to ensure everything continues to run smoothly? Who covers for you?
    4. What tools or structures are in place to help you work at the top of your license?
    5. What training did you receive to help you to take on your new roles/duties?
12. Why do you think this clinic was selected for the LEAP Project? (What do you think is innovative/creative about the way you work?)
    1. Based on the conversations we have had with others at this clinic thus far, we understand other key innovations in primary care delivery are ... <fill in description>, is there anything you would want to clarify or add to this description?
    2. [If not clear] What are the key roles and work processes in this innovation?
13. Can you describe how patients experience care at your site?
    1. <Insert brief standardized care vignette here. Ask how their practice would approach caring for this patient and to describe their particular role in that>
    2. How is the care provided to complex patients different from patients in relatively good health?
    3. What unique services do your patients receive?
14. [IF NOT ALREADY COVERED IN HISTORY]Can you tell me about the history of your innovation(s)? How did it come about?
    1. [IF NOT ALREADY COVERED IN HISTORY]What were the key motivations for making these changes?
15. [IF NOT ALREADY COVERED IN HISTORY]What aspects of your organization had to change to accommodate these changes? <Or if organization is young and was started with innovation in place> What did you need to do differently in order to make these innovations possible?
16. What tools have you created to support this innovation in your practice/support your primary care teams? (care tools, training, work flows, QI, team building activities)
    1. [If org chart available—might want to ask about specific roles]
17. Can you please describe any other factors that you feel supported this change?
    1. Participation in collaborative?
    2. Financial incentives? Grants?
    3. Support from a parent organization or business partner?
    4. Partnerships with other community resources?
    5. Has the physical environment of the clinic changed to accommodate your innovations? If so, how?
18. Can you please describe the barriers you encountered to put this innovation in place?
    1. State policies?
    2. Reimbursement issues?
    3. Union or scope of work issues with clinical staff?
    4. Hiring, training, and retention?
    5. Patient demands?
    6. How did you address these barriers?
    7. Barriers created by a parent organization or business partner?
19. Can you talk about if and how your organization has accessed and/or partnered with other organizations in community?
20. **Innovation—Short/Intermediate Outcomes**
21. Can you tell me about the impact the innovation has had on your practice?

[If not already addressed earlier, touch on staff work environment]

- 1. How would you describe the overall culture or atmosphere in <the site visit clinic>?
  2. What is it like working here?
  3. How would you describe your relationships with your coworkers?
  4. What is the best thing about work here?
  5. What is the most challenging thing about working here?

1. Can you tell me how your innovative care model affected your relationships with your patients?
   1. Can you provide a story that provides an example of how you work with a patient differently because of this innovation (or “your role” if it is central to the innovation), compared to the way this practice did things before?
   2. What impact has the [specific workforce components that changed] had on patients?
   3. What feedback have you received from patients about your innovative care model? [consider asking about each specific component’s impact on pts]
   4. Can you describe a specific incident or occasion that you feel is representative of the kind of feedback you get patients?
   5. What mechanism(s) do you have for collecting feedback from patients?
   6. How is this feedback shared with staff?
2. How sustainable is this care model for your organization?
   1. What do you think is important for sustaining this model?
   2. What challenges are you most concerned about for the future of this model?
   3. Do you have any indication how this innovation may help you control costs?
3. What do you see for your organization in 3 years from now?
   1. What are the 2-3 things are you most stuck on? <thinking to the Learning Community>
4. Tell me about some of the lessons learned that you would want to share with other practices seeking to implement this kind of innovative model.
   1. Anything you would have done differently?
   2. Any surprises along the way?

**PHOTOWORK – give staff member:**

- **PhotoWork guidelines**
- **A digital camera, or a disposable camera <have them write their name on it>**
- **envelopes for shipping back to us – one padded and one large self addressed stamped**
- **Photo Release forms, if they photograph anyone besides a fellow staff member**
