# Supplementary material for: Effective team-based primary care: observations from innovative practices
Source: BMC Fam Pract. 2017 Feb 2;18:13. doi: 10.1186/s12875-017-0590-8 (PMC5289007; doi:10.1186/s12875-017-0590-8)
Supplement: Additional file 2: — LEAP Interview Guide – Leadership. Guide for conducting recorded group interviews with administrative and clinical leaders in each visited clinic. (DOCX 22 kb) [file 12875_2017_590_MOESM2_ESM.docx]

**PCT-LEAP Site Visit Interview Guide**

**Leadership (Group Interview – 2 hours)**

04-22-13

2 hours

*Thank you all for participating in this interview. Any questions about this interview or the LEAP Project?*

<Collect signed consent form – not a research study but we do want to publish>

1. **Role**
2. Can you (all) please say your title and briefly describe your role within <org name>?
   1. How long have you been at this practice?
   2. What relevant background did you have before coming to this practice?
3. **Care teams/staffing**
4. We would like to understand how you build your teams. What is your current staffing model? (How do you link staffing to patient panels? How are care teams organized - who is the primary care team of the patient at the most basic level? Who is on the extended care team?)
   1. <Might want to ask about specific roles>
   2. What, if any, staffing do you have to provide care such as behavioral health, nutrition, diabetes education, case management, etc?
   3. How do you staff and finance care coordination?
   4. How would you expand your staffing model?
   5. Are there any other unique features of your care teams that we should be aware of?
   6. How do you ensure that your staff are working at the top of their license?
   7. What tools, training or guidelines do you find you need to have in place to ensure that people are working at the top of their license?
   8. How do you orient and train staff members in their role?
   9. What challenges, if any, have you had recruiting and retaining staff?
   10. Have you had problems with staff turnover?
5. **Organization History/Mission/Evolution**
6. Can you take a few minutes to briefly describe your organization’s history?
   1. What key turning points/milestones in your organization’s history do you believe are critical to understanding the organization now?
   2. How long has your organization been in existence? (When did this specific clinic open?)
   3. How would you describe the leadership structure? (clinical medical leadership: is there a CMO? ACMO? CNO? Chief of behavioral health? Do all medical staff report through the CMO and then to CEO?)
   4. Can you tell me how are key strategic decisions made? (growth, change in staffing patterns, or change in services)
   5. As leaders do you have a particular approach or philosophy when it comes to leadership? If so, what is it and how does it play out in your day-to-day management?
   6. Is there anyone who has left the organization that was particularly important to its history and/or the development of the innovations we are interested in? If so, who was that person and what role did they play in making the organization what it is today?
   7. How did you build your practice, in terms of key components like empanelment, access, population management, data-driven improvement, etc? <building blocks>
7. Can you please describe your organization’s mission?
8. What are the overarching goals or principles that drive major strategic decisions?
9. When did your organization implement an electronic medical record (if not implemented-Do you plan to implement and when)?
   1. What, if any, challenges did you have transitioning to an EMR?
   2. Do you offer an online patient portal? How is that going? (are pts using it? What functions does it offer? How has it changed interactions w pts?)
10. Please tell me about any unique aspects of your financial model that we should be aware of, such as finding alternate sources of funding, grants, charging monthly membership fees, etc?
    1. Are you NCQA or Joint Commission recognized as medical home—since when?
    2. What kind of financial metrics do you track? How much does it cost for you to see a pt under your current model? (Can you tell us the pmpm?)
11. How would you describe your target population(s)?
    1. [If the org’s mission is to serve a specific population] How has your organization stayed responsive to the needs of this population over time?
12. What other community or contextual factors do you think have been critical to shaping <org name>?
    1. What other types of support for your work are available in your community?
    2. What unique challenges do you face in this community?
    3. How does your state’s Medicaid program impact your practice?
    4. Please tell me about any specific issues in your state or community that impact billing and reimbursement.
    5. How do state policy impact staffing (such as scope of practice restrictions, restrictions on MA practice)?
13. Can you talk about if and how your organization has accessed and/or partnered with other organizations in community?
14. Can you describe how patients experience care at your site?
    1. How is the care provided to complex patients different from patients in relatively good health?
    2. What unique services do your patients receive?
15. What is your organization’s approach to quality improvement – or another way to think about what we are getting at, how do you *make change*?
    1. Do you have a particular model for QI or change management that you follow? (Lean, Six Sigma, etc)
    2. What structures are in place to support QI efforts?
    3. Can you describe your performance improvement plan? <if necessary> Do you have a written performance improvement plan?
    4. How are staff members involved in QI?
    5. Can you give us an example of how your QI/change management approaches relate to successes in your workforce/staffing model?
    6. You have given us many of the measures you use, is there anything else in particular you think we should know about those measures and/or how you use them?
16. **Primary Care Workforce Innovation**
17. Thank you for talking to us in previous conversations about what you have done that is innovative. Can you please choose one key workforce innovation and we will talk about that a little more in depth.
    1. [If not clear] What are the key roles and work processes in this innovation?
    2. How do you innovate around patient safety?

**<Repeat Q14-19 if needed about specific innovations>**

1. [IF NOT ALREADY COVERED]What is the history of your innovation? How did it come about?
2. [IF NOT ALREADY COVERED]What were the key motivations for making these changes?
3. [IF NOT ALREADY COVERED]What aspects of your organization had to change to accommodate these changes? <Or if organization is young and was started with innovation in place> What did you need to do differently in order to make these innovations possible?
   1. How do you pay for it?
   2. How do you compensate staff who have different responsibilities compared to local averages for those roles traditionally?
4. What tools have you created to support this innovation in your practice/support your care teams? (for training/orientation, after visit summaries, standing orders, QI, team building activities)
5. Can you please describe any other factors that you feel supported this change?
   1. Participation in collaborative?
   2. Financial incentives?
   3. Support from a parent organization or business partner?
   4. Partnerships with other community resources?
   5. Has the physical environment of the clinic changed to accommodate your innovations? How?
6. Can you please describe the barriers you encountered?
   1. State policies?
   2. Reimbursement issues?
   3. Union or scope of work issues with clinical staff?
   4. Hiring, training, and retention?
   5. Patient demands?
   6. Barriers created by a parent organization or business partner?
   7. How did you address these barriers?
7. **Innovation—Short/Intermediate Outcomes**
8. Can you tell me about the impact the innovation has had on your practice?

[If not already addressed earlier, touch on staff work environment]

- 1. How would you describe the overall culture or atmosphere in <the site visit clinic>?
  2. What is it like working here?
  3. How would you describe your relationships with your coworkers?
  4. What is the best thing about work here?
  5. What is the most challenging thing about working here?

1. Can you tell me how your innovative care model affected your relationships with your patients?
   1. Can you provide a story that provides an example of how you work with a patient differently because of this innovation (or “your role” if it is central to the innovation), compared to the way this practice did things before?
   2. What impact has the [specific workforce components that changed] had on patients?
   3. What feedback have you received from patients about your innovative care model? [consider asking about each specific component’s impact on pts]
   4. Can you describe a specific incident or occasion that you feel is representative of the kind of feedback you get patients?
   5. What mechanism(s) do you have for collecting feedback from patients?
   6. How is this feedback shared with staff?
2. How sustainable is this care model for your organization?
   1. What do you think is important for sustaining this model?
   2. What challenges are you most concerned about for the future of this model?
   3. Do you have any indication how this innovation may help you control costs?
3. What do you see for your organization in 3 years from now?
   1. What are the 2-3 things you are most stuck on? <thinking to the Learning Community>
4. Tell me about some of the lessons learned that you would want to share with other practices seeking to implement this kind of innovative workforce model.
   1. Anything you would have done differently?
   2. Any surprises along the way?
5. If you were to leave here and go to another practice, what 3 things would you take with you?
