# Supplementary material for: Effective team-based primary care: observations from innovative practices
Source: BMC Fam Pract. 2017 Feb 2;18:13. doi: 10.1186/s12875-017-0590-8 (PMC5289007; doi:10.1186/s12875-017-0590-8)
Supplement: Additional file 3: — PCT LEAP Site Visit Observation Guide – Clinical Expert. The guide includes instructions as to what clinical site visitors should observe and document during site visits. (DOC 125 kb) [file 12875_2017_590_MOESM3_ESM.doc]

1. ***INSTRUCTIONS***

- *Your main objective is to observe care in action. Focus on capturing and helping SV team understand:*

1. ***What is unique and sets them apart from traditional practices?***
2. ***Are they doing what they said they are doing? What specific examples did you observe (care processes, work flows, role distribution)?***

- *This is a quick reference for your observations/notes, organized by the Case Summary outline*

***ii. YOUR SCHEDULE***

You have flexibility with your time and activities; the SV schedule provides suggestions.

| **Clinical Expert Role** |
| --- |
| - **Guidance on clinical relevance** - **Participate in SV team debriefs**, help team contextualize observations - Build relationships with leaders - Leadership dinner, if possible - Tour - Lead EMR demo <guide below> - Leadership interview; optional at all others - Informal observations, conversations, staff shadowing (focus on providers & key roles) - Take photos - Observe huddles and other key meetings - Identify tools to collect |

**EMR Demo Protocol**

**You lead the conversation**. Probes:

1. What are the most helpful features of your EMR?
   1. Can you show me what you find most helpful?
   2. Can you show me what makes it easier for people here to do their work?
2. What are the population management features of your EMR & how do you use them? Who uses them the most?
3. Do your patients have access to their EMR?
   1. If yes, how has that changed the work that you do?
4. What were the biggest work process/flow changes you have made to accommodate your EMR and how did you accomplish them?
   1. How has the EMR influenced staffing roles and responsibilities?
5. Does your EMR facilitate provider-to-provider communication? Secure messaging, referral mgmt, etc
6. What do you wish you could do with your EMR/health IT but cannot do yet and why?
7. Do you offer an online patient portal? How is that going? (are pts using it? What functions does it offer? How has it changed interactions w pts?)

***RATINGS***

Overall:

|  | Strongly Disagree | Disagree | Neutral | Agree | Strongly Agree |
| --- | --- | --- | --- | --- | --- |
| Workforce is truly innovative | 1 | 2 | 3 | 4 | 5 |
| Staff heedfully interrelate | 1 | 2 | 3 | 4 | 5 |
| Strong on adaptive leadership | 1 | 2 | 3 | 4 | 5 |
| Use data to make things better | 1 | 2 | 3 | 4 | 5 |
| Meeting needs of community/pt popln | 1 | 2 | 3 | 4 | 5 |
| **I would want to be a patient here** | **1** | **2** | **3** | **4** | **5** |

**Team culture/clinic functioning:** communications, trust, wk environ

|  | Strongly Disagree | Disagree | Neutral | Agree | Strongly Agree |
| --- | --- | --- | --- | --- | --- |
| Learn from each other | 1 | 2 | 3 | 4 | 5 |
| Learn new things together as a group | 1 | 2 | 3 | 4 | 5 |
| Problem solve together | 1 | 2 | 3 | 4 | 5 |
| Staff understand their role | 1 | 2 | 3 | 4 | 5 |
| Staff connect their work to the big picture | 1 | 2 | 3 | 4 | 5 |
| Trust among team members | 1 | 2 | 3 | 4 | 5 |
| Overly chaotic/dysfunctional | 1 | 2 | 3 | 4 | 5 |
| Leadership creates an environment where things can be accomplished | 1 | 2 | 3 | 4 | 5 |

**Exit protocol: “Debrief” with site leaders**

1. **List the key workforce innovations we saw**, and provide high level positive feedback
2. LEAP next steps to expect:
   1. Follow-up w qualitative researcher on tools and clarifications/help with things we missed.
   2. Review case summary in the next 2-4 months, a descriptive not evaluative summary.
   3. Will not receive critical feedback/advice from us; will get from true experts, the other exemplars
3. Learning Community invite
   1. Describe current vision
   2. Solicit feedback to inform LC development (+ what 3 things they are struggling with most)
4. ***OVERALL IMPRESSIONS, summary of Workforce innovations***

- Identify key workforce innovations
- What struck you as the most innovative/exciting aspects of this practice? **(specific examples)**
- How does it go beyond a good traditional practice? **(specific examples)**

1. **CONTEXT AND HISTORY (external/community and internal/organizational descriptions)**

- External contextual factors
- Community
- Patient population
- Organizational History – how did they get there? Milestones, key factors in practice transformation
- Health IT, EMR implementation
- Leadership – structure, style/approach
- Internal Culture – overall feel of the practice/general work environment

1. ***STAFFING and activities***

- Care Team Model (core primary care team; centralized; consultative; admin/off-stage staff)
- List roles and functions of staff members starting from core going outward.
- Interactions
- Innovative roles, workflows

**MATRIX** of staff roles, functions *–placeholder, to be added*

1. ***BUILDING TEAMS***

- Hiring and Training
- Teamwork and Communication
  - key structural supports: team meetings, physical environment/collocation, etc
  - culture of heedful interrelating, listening, trust, etc
- Lessons learned

1. ***quality improvement***

- How collect and use data
  - Metrics tracked: quality, health outcomes, cost/ sustainability, pt perspective, staff wk environ
  - How use it, when, by whom (reports/run charts, popln and/or panel level, QI, pay for performance)
- Approach to change management
- Involvement of staff at all levels

1. ***Vignettes***

- Stories that highlight the patient perspective and care team interactions

*bonus prize* if yours is selected for the first case summary ☺

1. ***change concepts***

Using SNMHI 8 change concepts, describe experience with:

- Engaged Leadership
- QI strategy
- Empanelment
- Continuous & Team-based Healing Relationships
- Patient-Centered Interactions
- Organized, Evidence-Based Care
- Enhanced Access
- Care Coordination

1. ***SUSTAINABILITY and next steps***

- Business/financial stability, controlled costs
- How do they pay for this?
- Sustainability of innovations
- Next steps

1. ***Tools***

- Collect tools developed to support their workforce innovations
- E.g., training curriculum, job descriptions, other templates used in hiring/orienting staff to roles, workflows, job aids, standing orders, patient care plan templates, after-visit summaries, QI plans, EMR protocol & templates
